# Anthrax Lethal Toxin-Induced Gene Expression Changes in Mouse Lung

**DOI:** 10.3390/toxins3091111

**Published:** 2011-09-07

**Authors:** Eric K. Dumas, Philip M. Cox, Charles O’Connor Fullenwider, Melissa Nguyen, Michael Centola, Mark Barton Frank, Igor Dozmorov, Judith A. James, A. Darise Farris

**Affiliations:** 1 Department of Microbiology and Immunology, University of Oklahoma Health Sciences Center, 1100 N. Lindsay, Oklahoma City, OK 73104, USA; Email: dumase@omrf.org (E.K.D.); melissa-nguyen@ouhsc.edu (M.N.); jamesj@omrf.org (J.A.J.); 2 Arthritis and Clinical Immunology Program, Oklahoma Medical Research Foundation; 825 NE 13^th^ Street, MS 53, Oklahoma City, OK 73104, USA; Email: coxp@omrf.org (P.M.C.); charles-fullenwider@ouhsc.edu (C.O.F.); centolam@omrf.org (M.C.); frankb@omrf.org (M.B.K.); dozmorovi@omrf.org (I.D.); 3 Microarray Research Facility, Oklahoma Medical Research Foundation, 825 NE 13th Street, MS 53, Oklahoma City, OK 73104, USA

**Keywords:** Lethal Toxin, lung, gene expression

## Abstract

A major virulence factor of *Bacillus anthracis* is the anthrax Lethal Toxin (LeTx), a bipartite toxin composed of Protective Antigen and Lethal Factor. Systemic administration of LeTx to laboratory animals leads to death associated with vascular leakage and pulmonary edema. In this study, we investigated whether systemic exposure of mice to LeTx would induce gene expression changes associated with vascular/capillary leakage in lung tissue. We observed enhanced susceptibility of A/J mice to death by systemic LeTx administration compared to the C57BL/6 strain. LeTx-induced groups of both up- and down-regulated genes were observed in mouse lungs 6 h after systemic administration of wild type toxin compared to lungs of mice exposed to an inactive mutant form of the toxin. Lungs of the less susceptible C57BL/6 strain showed 80% fewer differentially expressed genes compared to lungs of the more sensitive A/J strain. Expression of genes known to regulate vascular permeability was modulated by LeTx in the lungs of the more susceptible A/J strain. Unexpectedly, the largest set of genes with altered expression was immune specific, characterized by the up-regulation of lymphoid genes and the down-regulation of myeloid genes. Transcripts encoding neutrophil chemoattractants, modulators of tumor regulation and angiogenesis were also differentially expressed in both mouse strains. These studies provide new directions for the investigation of vascular leakage and pulmonary edema induced by anthrax LeTx.

## 1. Introduction

*Bacillus anthracis* is a spore-forming, gram-positive bacterium that is the causative agent of anthrax. Use of *B. anthracis* spores as a bioterror weapon in the United States in 2001 has renewed interest in exploring the mechanisms of pathogenesis and mortality induced by *B. anthracis* infection. At least three forms of anthrax are known, are defined by the route of spore entry, and are characterized by differing rates of mortality. Among these, the inhalation form is the most deadly, with a mortality rate of at least 40% even with a high suspicion of *B. anthracis* and the best available medical care [1]. The extraordinarily high mortality of the inhalation route is likely a consequence of the ensuing bacteremia that is an invariable consequence of this route of infection.

One of the major virulence factors of *B. anthracis* is its tripartite toxin, composed of Protective Antigen (PA), Lethal Factor (LF) and Edema Factor (EF) [2,3]. PA and LF combine to form Lethal Toxin (LeTx), while PA and EF combine to form Edema Toxin (EdTx). PA is the receptor binding component for both toxins. Following binding to one of its cell surface receptors, tumor endothelial marker 8 (TEM8) or capillary morphogenesis protein 2 (CMG2), PA is cleaved by cell-membrane bound furin-like proteases, resulting in 63 kD and 20 kD PA fragments. Following cleavage, the PA_63_ subunits heptamerize, forming a prepore, and bind up to three molecules of LF and/or EF. The prepore is then endocytosed and, upon acidification of the endosome, undergoes a conformational change becoming the PA pore, allowing for LF and/or EF entrance into the cytoplasm. 

As its name suggests, LeTx, a Zn^2+^ metallo-protease that cleaves Mitogen Activated Protein Kinase Kinases (MAPKKs) [4], is lethal when delivered to laboratory animals by the systemic route [5,6,7]. The toxicity of LeTx is not considered high in comparison with other bacterial toxins [8]. However, during the peak of the bacteremic stage of infection, the load of *B. anthracis* vegetative bacteria is known to grow to 10^8^ organisms/mL of blood, resulting in levels of secreted LF in the lethal range. Indeed, after a critical threshold of bacteremia is achieved, even effective sterilization of the blood by appropriate antibiotic treatment fails to curb mortality [9]. The capacity of *B. anthracis* to reach such high bacteremic levels is thought to be a consequence of direct effects of LF on immune cells early in infection. LF exposure leads to macrophage and dendritic cell death [10], inhibits T and B lymphocyte responsiveness [11,12], and impairs chemotaxis of neutrophils [13].

Systemic administration of lethal doses of LeTx to laboratory animals is known to cause blood vessel leakage, pulmonary edema and cytokine-independent vascular collapse [5,6,7]; however, the molecular mechanisms leading to these terminal events remain unclear. While a slight cytotoxic effect of LeTx on lung endothelial cells has been observed *in vitro,* endothelial cell death was not responsible for LeTx-induced barrier dysfunction [14]. Moreover, *in vivo* evidence of endothelial cell death was lacking in mice following administration of lethal LeTx doses [5].

The hypothesis underlying this study was that global gene expression analysis of a highly vascularized tissue known to be affected by anthrax LeTx would reveal LeTx-specific changes in the expression of genes whose products are known to be associated with vascular and/or capillary leakage in other settings. In keeping with this hypothesis, analysis of global gene expression in lung tissue of mice at an early time point following systemic exposure to LeTx has resulted in the identification of new candidate molecular pathways that may promote LeTx-induced vascular effects.

## 2. Materials and Methods

### 2.1. Mice

A/J and C57BL/6J (B6) six-week-old female mice were purchased from Jackson Laboratories (Bar Harbor, ME, USA) and housed in the Laboratory Animal Resource Center at Oklahoma Medical Research Foundation (OMRF). At this age, the body weights of these two strains are indistinguishable [15]. All mouse experiments were approved by the OMRF Institutional Animal Care and Use Committee. Mice were acclimated to the facility for one week prior to experimentation. 

### 2.2. Lethal Toxin and Mutant Toxin

For the LD_50_ study, recombinant PA and LF were produced in *E. coli* as previously described [16,17]. rLF and rPA proteins were produced as amino-terminal His_6_-tagged proteins. Cultures of BL-21/pET15b (containing LF or PA cDNA from *B. anthracis*) were grown in Luria broth containing ampicillin (50 µg/mL) to an optical density at 600 nm (OD_600_) of 0.6 to 0.8. Protein expression was induced by addition of 1 mM isopropyl β-D-thiogalactoside (IPTG) and incubation overnight at 16 °C. Cells were then pelleted and disrupted with a French press. The lysed cells were then centrifuged, and the supernatant was passed over a Ni^2+^-charged column equilibrated with a binding buffer (Novagen, Gibbstown, NJ, USA). The bound fusion protein was removed with 0.5 M imidazole according to the manufacturer's instructions (Novagen). The eluted protein was then de-salted by passage through a PD-10 column (GE Healthcare, Piscataway, NJ, USA). Protein concentration was determined by a standard Bradford assay (Bio-Rad, Hercules, CA, USA) and stored at 4 °C on ice. Purified PA and LF (20 µg) were electrophoresed in a 12.5% sodium dodecyl sulfate-polyacrylamide gel and visualized by Coomassie blue staining to ensure purity. For global gene expression studies, standardized, quality controlled preparations of PA, LF and an inactive mutant form of LF (E687C) were all purchased from List Biological Laboratories (Campbell, CA, USA). The manufacturer’s reported endotoxin levels in the wild type PA and wild type LF preparations were 0.00069 and 0.00255 EU/µg, respectively. Endotoxin levels of the mutant LF preparation were unavailable.

### 2.3. Injections and Sampling

For the LD_50_ study, PA and LF (LeTx) at an approximate ratio of 7:3 w:w was administered intraperitoneally in 0.2 mL/mouse PBS pH 8.0 at various concentrations to groups (*N* = 6 mice/group) of mice and deaths recorded twice daily for 7 days. For gene expression studies, PA, LF and E687C mutant LF were reconstituted according to the manufacturer’s instructions. Preparations of mixed PA plus LF (LeTx) or mixed PA plus E687C mutant LF (Mutant Tx) diluted with PBS pH 8.0 were then administered to groups (4 mice per group) of mice as described above except that the dosage per mouse was 120 µg PA + 50 µg LF for LeTx or 120 µg PA + 50 µg E687C mutant LF for Mutant Tx delivered in a volume of 0.25 mL per mouse. The mice were euthanized 6 hours later and lung tissue collected. Lung lobes from one side of the thoracic cavity were removed and placed in 1 mL of TRIZOL for RNA isolation. Remaining lobes were removed, fixed in formalin and paraffin blocked. Sections (6 µM) were stained with hematoxylin and eosin and examined for cellular infiltrates.

### 2.4. RNA Isolation and evaluation of Global Gene Expression

All surfaces were sprayed and wiped with RNaseAway− to prevent RNase contamination. All tissues were homogenized in 1 mL of TRIZOL using a Tissue-Tearer (BioSpec Products, Bartlesville, OK, USA). The RNA was then isolated using the RNeasy Mini Kit from QIAGEN following the kit protocol. RNA quality was verified using an Agilent 2100 Bioanalyzer and concentrations determined by NanoDrop 1000 spectrophotometry. RNA samples were labeled using the Illumina Total Prep RNA Amplification Kit following manufacturer’s directions (Ambion; Austin, TX, USA). Briefly, cDNA was reverse transcribed from RNA after priming with T7-oligo-dT, and cRNA was synthesized *in vitro* from the T7 promoter while incorporating biotinylated UTP. cRNA was hybridized overnight to 25 K Illumina MouseRef-8 v. 1.1 Expression BeadChips for global gene expression. Microarray chips were washed to high stringency and labeled with streptavidin-Cy3 (Amersham Biosciences; Piscataway, NJ, USA) prior to scanning on an Illumina BeadArray Reader.

### 2.5. Data Analysis

For LD_50_ determinations, percent death was plotted as a function of either PA or LF concentration. Interpolation of the concentration of PA and LF at the 50% death point was performed in Microsoft Excel. LD_50_ values reported are based on analysis of LF plots.

Analysis of the gene expression data was conducted using an internal standard-based method described in detail elsewhere [18,19,20,21]. This method has been used recently by several groups to identify differentially expressed genes from whole genome array data with high sensitivity and specificity [22,23,24]. The method involves a two-step normalization procedure and a three-step Associative analysis. Briefly, the first step is defining the average and standard deviation (SD) of the background for each array and transforming the data such that these values are 0 and 1, respectively. After this transformation the gene expression data are presented in “normalized units” (NU), with 1 NU = 1 SD of the background distribution. Based on this determination, the threshold of 3 SD above the mean of background is used to preliminarily distinguish between expressed and non-expressed genes. In the second step of the normalization procedure, robust linear regression is applied to a homogeneous set of equally expressed genes to normalize arrays to each other. Next, differential expression is determined using a three-step Associative analysis. In the first of these procedures, a “reference group” of biologically stable genes is constructed (comprised of several thousands of members) that are expressed above background but with low inherent variability as determined by an *F*-test. This reference group is used as an internal standard of equity of expression. The second procedure in this part of the method applies a Student’s *T*-test using the commonly accepted threshold of *p* < 0.05 to obtain a preliminary list of differentially expressed genes that will include false positives determinations. The third step applies an Associative *T*-test in which the replicated residuals for each gene from the experimental group are compared with the entire set of residuals from the reference group of genes, defined above. Thus, the Associative *T*-test tests the null hypothesis that the levels of gene expression in the experimental group (presented as deviations from the averaged control group profile) is associated with a highly representative, normally distributed set of residuals of gene expression values in the reference group. The Associative significance threshold is then adjusted to render the appearance of false positive determinations improbable [21]. Only genes that pass both the standard and Associative *T*-tests are defined as differentially expressed. The thus-defined differentially expressed genes reported herein exhibited minimum average expression levels of 20 times above background. We report minimum 1.5 fold differences in order to concentrate attention on the reproducible changes of likely biologic significance. The use of 4 replicates per group in this study is based upon power analyses reported elsewhere [18,21]. The two-step normalization procedure and the Associative analysis functions are implemented in MatLab (Mathworks, MA, USA) and are available from the authors upon request. These algorithms are also obtainable from an R package diffGeneAnalysis, available as part of Bioconductor packages [25]. 

Ingenuity Pathways Analysis was used for classification of genes into physiologic categories (Ingenuity Systems, Redwood City, CA, USA).

## 3. Results

### 3.1. Differential Susceptibility of A/J and C57BL/6 Mice to Death Induced by Anthrax LeTx

**Figure 1:**
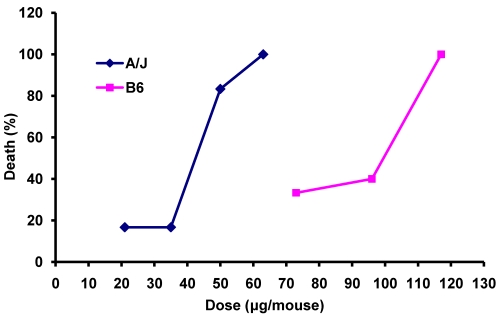
Relative susceptibility of A/J and B6 mice to death by systemic exposure to anthrax LeTx. Percent death in groups of 6 mice per dosage point in terms of amount of LF delivered per mouse is shown. PA and LF were delivered *i.p.* at ratios of 7:3 w:w. Dosage ranges for each strain were determined in preliminary experiments.

Susceptibility of various inbred strains of mice to death from LeTx varies and does not correlate with susceptibility of their isolated macrophages to necrotic death *in vitro* [26]. While LeTx-induced necrotic death of murine macrophages is associated with polymorphisms in the *Nalp1b* gene [27], mechanisms of genetic control of mouse death by LeTx are unclear. Macrophages of both A/J and C57BL/6 (B6) mice are susceptible to LeTx-induced apoptosis but resistant to LeTx-induced necrosis *in vitro* [26]. To determine whether these two strains harbor differences in susceptibility to whole animal death by systemic administration of LeTx, we challenged groups of A/J and B6 mice with various doses of LeTx and recorded deaths over a 7 days period. We observed that B6 mice were less susceptible to LeTx-induced death than mice of the A/J strain (Figure 1). The LD_50_ of LeTx delivered in the same ratio thought to infect cells (7:3 PA:LF) was calculated as 100 µg PA/43 µg LF and 215 µg PA/ 92 µg LF for the A/J and B6 strains, respectively. Thus, an approximate 2-fold difference in LD_50_ was measurable in these two strains. A previous study that found similar susceptibility to LeTx-induced death in these two strains evaluated only a single concentration of PA and LF and delivered the proteins at a 1:1 ratio [26].

### 3.2. Lack of Obvious Pulmonary Pathology in Mice Receiving Systemic Exposure to Anthrax LeTx

**Figure 2:**
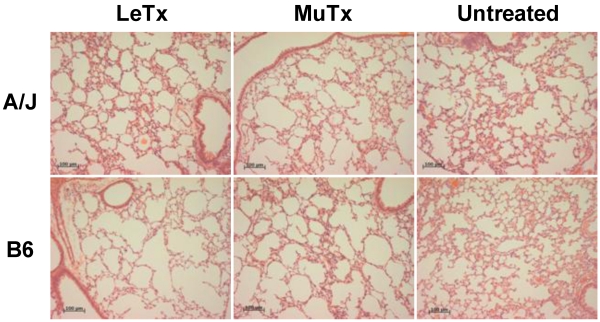
Normal histology and absence of immune cell infiltrates in mouse lungs 6 h following systemic exposure to anthrax Lethal Toxin (LeTx) or inactive mutant toxin (MuTx). Representative lung sections stained with hematoxylin and eosin showing normal lung architecture and absence of immune cell infiltrates or other pathologic findings. Similar results were observed 12 h following LeTx treatment. Bars mark 100 µm.

A previous pathophysiologic and histologic study of B6 and BALB/c mice systemically treated with LeTx demonstrated 5–10 fold increases in circulating neutrophils in both strains; however, no tissues revealed evidence of neutrophil margination in blood vessels even at late time points [7]. Moreover, lungs remained free of inflammatory cells, though the mice suffered from extensive pleural fluid accumulation between 48 and 60 h post-exposure. Histologic evaluation of lung tissue sections at early time points (6 and 12 h post-injection) following *i.p.* injection of LeTx and an inactive mutant form of LeTx in the present study revealed normal-appearing lung tissue in both strains with no evidence of immune cell infiltrates and normal tissue architecture, as expected (Figure 2).

### 3.3. LeTx-Specific Gene Expression Changes in Mouse Lung Include Genes that are Candidates for Mediating or Promoting Vascular Leakage and Pulmonary Edema

**Figure 3:**
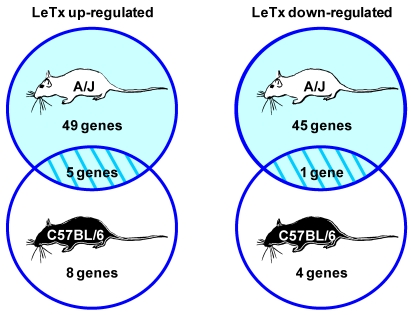
Number and correspondence of early LeTx-specific gene expression changes in A/J and C57BL/6 mouse lungs. Diagrams depict within-strain comparisons of gene expression in wild type LeTx- versus Mutant Tx-treated mice following treatment. Differential gene expression was assessed using 4 mice per group. Only genes exhibiting ≥1.5 fold differences were considered.

All gene expression data discussed in this publication have been deposited in NCBI’s Gene Expression Omnibus [28] and are accessible through GEO series accession number GSE28554 [29]. In total, 100 genes were differentially expressed ≥ 1.5 fold in A/J mice treated with wild type or mutant toxin (Table 1), while 18 genes were differentially expressed in B6 mice treated with wild type or mutant toxin (Table 2). Thus, significantly fewer genes (80% fewer) were modulated in the B6 strain compared to the A/J strain (compare Table 1 and Table 2), consistent with reduced susceptibility of B6 mice to LeTx-induced death. Genes that were up-regulated in both strains included *IGKV6-15*, *IGKV6-25*, *IGKV1-117*, *Tet1* and *Jakmip1*. *Tmem2*, a gene encoding a transmembrane protein of unknown function, was commonly down-regulated in both strains of mice (Figure 3).

**Table 1:** Most significant expression differences in lungs of LeTx-treated A/J mice compared to Mutant Tx-treated A/J mice. A subset of significantly differentially expressed genes is shown based on the following criteria: (i) Average expression values must equal or exceed 20 Normalized Units and (ii) magnitude of differences must be ≥1.5-fold. (**A**) Wild Type LeTx (**B**) Mutant Tx (**C**) Fold change LeTx/Mutant Tx. * Average expression in Normalized Units.

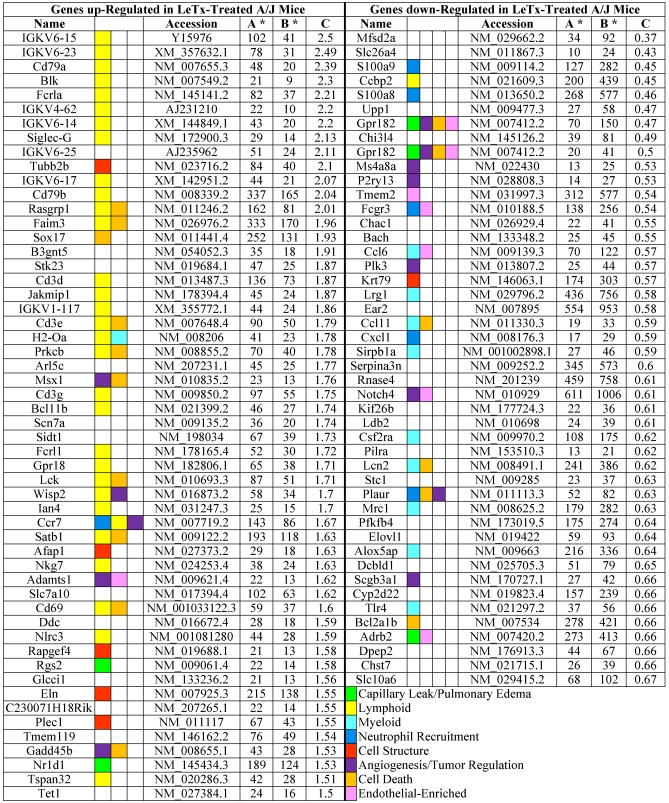

**Table 2:** Most significant expression differences in lungs of LeTx-treated B6 mice compared to Mutant Tx-treated B6 mice. A subset of significantly differentially expressed genes in B6 mice is shown based on the following criteria: (i) Average expression values must equal or exceed 20 Normalized Units and (ii) magnitude of differences must be ≥1.5-fold. * Average expression in Normalized Units.

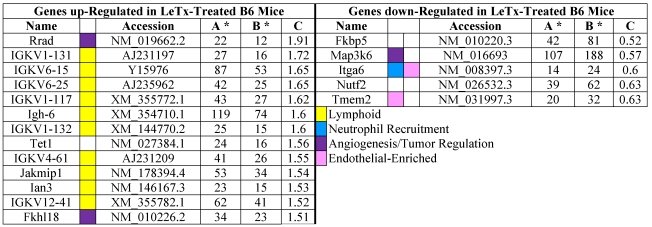

At least eight of the differentially expressed transcripts are normally enriched in endothelial cells [30,31]. These included *Notch4*, *Gpr182*, *Adrb2*, *Ccl6*, *Fcgr3*, *Tmem2*, *Itga6*, and *Adamts1*. Furthermore, at least three LeTx-induced gene expression changes that could promote vascular/capillary leakage were observed in the lungs of A/J mice. Modified expression of two endothelial-enriched genes, *Gpr182* and *Adrb2*, may also be associated with vascular/capillary leakage. We found the expression of *Gpr182*, (G-protein coupled receptor 182), a gene encoding a putative receptor for a vasoactive peptide hormone that has protective effects on both endothelium [32,33] and in lungs [12], and *Adrb2* (Adrenergic β receptor 2), a known participant in alveolar fluid clearance [34,35], to be down-regulated in our study. A third differentially expressed, yet up-regulated transcript that may influence vascular permeability is *Rgs2* (Regulator of G-protein Signaling 2), which promotes cGMP-mediated vascular relaxation through G-protein coupled receptors [36].

The modulation of genes related to cell death and apoptosis, specifically in endothelial cells, could also contribute to vascular/capillary leakage [37,38]. Moreover, differentially expressed genes in this category might reflect LeTx-mediated death of other cell types such as resident myeloid cells. In lungs of A/J mice treated with wild type LeTx or its inactive mutant, the differential expression of 15 genes associated with cell death was observed. Expression of *Rasgrp1*, *Faim3*, *Sox17*, *Cd3e*, *Prkcb*, *Msx1*, *Lck*, *Satb1*, *Cd69* and *Gadd45b* was up-regulated while expression of *Gpr 182*, *Cxcl11*, *Lcn2*, *Plaur* and *Bcl2a1b* (B-cell leukemia/lymphoma 2 related protein A1b), the product of which protects macrophages from apoptosis [39], was down-regulated.

Actins, myosins, microtubule components and other gene products that control endothelial cell architecture are associated with vascular leakage [40] and have been observed at high levels in LeTx stressed endothelial cells *in vitro* [14]. Consistent with this, we observed that the expression of at least 6 such genes was specifically modulated in A/J lungs by wild type LeTx. These included the up-regulation of *Tubb2b*, *Eln*, *Rapgef4*, *Afap1*, *Plec1* and the down-regulation of *Krt79*.

Recent literature linking LeTx with cancer [32,41,42,43] prompted the categorization of genes associated with tumor regulation/angiogenesis. In A/J mice treated with wild type LeTx, *Ms4a8a*, *P2ry13*, *Plk3*, *Notch4*, *Plaur*, *Scgb3a1*, *Gadd45b*, *Ccr7*, *Wisp2*, *Msx1* and *Adamts1* were all differentially expressed, while in B6 mice injected with wild type LeTx, the expression of *Rrad*, *Fkhl18* and *Map3k6* was modulated.

Another modulated gene signature included the LeTx-mediated down-regulation of several genes encoding products that act as neutrophil chemoattractants. In A/J mice treated with wild type LeTx, these included *S100a8*, *S100a9*, *Fcgr3* and *Cxcl1*. Additionally, *Plaur*, which encodes CD87/uPAR, a receptor that has been shown to be critical for neutrophil recruitment *in vitro* [44] and for neutrophil recruitment into mouse lungs *in vivo* in bacterial infection models [45,46], was down-regulated in LeTx-treated A/J lungs. In B6 mice, *Itga6* was down-regulated by wild type LeTx. The product of this gene is an integrin component that is associated with chemotaxis of neutrophils [47]. One gene of this category, *Ccr7*, was up-regulated in A/J mice.

Surprisingly, the most dominant class of regulated genes observed in this study is expressed within lymphocytes and includes LeTx-mediated increases in expression of several transcripts encoding canonical components of T and B lymphocyte receptors. This class of genes accounted for over 25% of all Le-Tx-specific gene expression changes in A/J mice and over half in B6 mice (Table 1 and Table 2). Consistent with the well-known capacity of LeTx to mediate cell death in monocytes, macrophages and dendritic cells, reduced expression of a number of myeloid genes, representing 10% of the total number of differentially expressed genes in A/J mice, was also observed. 

Input of all differentially regulated transcripts listed in Table 1 into Ingenuity Pathways Analysis confirmed significant enrichment of genes involved in the processes of cellular apoptosis(*p* = 1.31 × 10^−5^), cellular movement (*p* = 8.77 × 10^−11^), quantity of lymphocytes (*p* = 2.91 × 10^−6^), activation of lymphocytes (*p* = 3.11 × 10^−6^) and chemotaxis of leukocytes (2.47 × 10^−12^) (Table 3). Interestingly, this analysis further revealed significant enrichment in genes related to uptake of arachadonic acid (*p* = 1.06 × 10^−4^) and orthostatic hypotension (*p* = 7.29 × 10^−4^) (Table 3). The number of differentially regulated genes in B6 mice (Table 2) was insufficient for Pathways analysis.

**Table 3:** LeTx-induced gene expression changes in A/J mice representing processes that are significantly enriched in comparison to all genes evaluated. Categorization of significantly enriched processes was determined by comparison to known associations within the Ingenuity Knowledge Base. Statistical significance was determined using a Fisher’s exact test with Benjamini-Hochberg correction for multiple testing.

	**Chemotaxis of Leukocytes (*p* = 2.47 × 10^−12^)**	**Quantity of Lymphocytes (*p* = 2.91 × 10^−6^)**	**Cellular Movement (*p* = 8.77 × 10^−11^)**	**Cellular Apoptosis (*p* = 1.31 ×10^−5^)**
**Up-Regulated**	Ccr7	Ccr7	Ccr7	Ccr7
	Eln	Cd3d	Cd3e	Cd3e
	Plec1	Cd3e	Cd69	Cd69
	Prkcb	Cd3g	Eln	Gadd45b
		Cd69	Lck	Lck
		Lck	Plec1	Msx1
		Rasgrp1	Prkcb	Prkcb
		Satb1	Rasgrp1	Rasgrp1
		Siglecg	Tubb2b	Satb1
			Wisp2	Sox17
				
**Down-Regulated**	Ccbp2	Bcl2a1b	Adrb2	Adrb2
	Ccl11	Notch4	Ccbp2	Bcl2a1b
	Chi3l4		Ccl11	Ccl11
	Csf2ra		Ccl6	Csf2ra
	Ear2		Chi3l4	Lcn2
	Plaur		Csf2ra	Plaur
	S100a8		Ear2	Serpina3n
	S100a9		Plaur	Tlr4
	Serpina3n		S100a8	
	Tlr4		S100a9	
			Serpina3n	
			Stc1	
			Tlr4	
				
	**Activation of Lymphocytes (*p* = 3.11 × 10^−6^)**	**Uptake of Arachidonic Acid (*p* = 1.06 × 10^−4^)**	**Orthostatic Hypotension (*p* = 7.29 × 10^−4^)**	
**Up-Regulated**	Cd3d		Ddc	
	Cd3e			
	Cd3g			
	Igkv1-117			
	Lck			
	Nlrc3			
	Prkcb			
	Satb1			
				
**Down-regulated**	Ccl11	S100a8	Adrb2	
	Tlr4	S100a9		

### 3.4. Gene Expression Differences that May Participate in Differential Susceptibility of A/J and B6 Strains to Death by LeTx

A large number of genes were differentially expressed between the A/J and B6 strains, consistent with expected genome-wide polymorphisms regulating global gene expression in these strains. As expected, most of these strain-determined differences in gene expression were irrelevant to LeTx treatment, as their expression levels were indistinguishable between the LeTx and Mutant Tx treated groups (data not shown). To find strain differences that may participate in the enhanced sensitivity of the A/J strain to death from LeTx treatment compared to the B6 strain, we identified the genes that exhibited both within-strain significant differential expression by wild type LeTx treatment compared to treatment with inactive mutant toxin (Table 1 and Table 2) and also significant differential expression between the strains treated with wild type LeTx. This list included 12 genes: three genes associated with tumor regulation/angiogenesis, two lymphoid cell-expressed genes, one myeloid cell-expressed gene and one endothelial-enriched gene (Table 4). One gene, *Elovl1*, appeared twice and represents differential expression measurements based on two different probes with this gene on the commercial gene microarray employed in this study.

**Table 4:** Strain differences in A/J mice (upper table) versus B6 mice (lower table) that are specific to LeTx treatment. Selections are listed from greatest to least magnitude of strain difference. *Average expression in Normalized Units. ** Selections follow the same trend in both strains and are therefore of less interest.

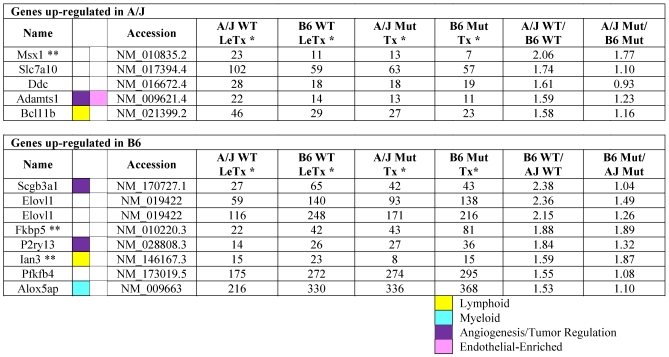

## 4. Discussion

Central features of both *B. anthracis*- and LeTx-induced animal death are vascular leakage and pulmonary edema. One important mechanism of LeTx-induced vascular leakage may be direct induction of endothelial cell death [37]. However, reports that LeTx-induced endothelial cell death is inefficient [38] and absence of measurable endothelial cell death in mice receiving LeTx doses sufficient to induce animal death [5] suggest that additional mechanisms may operate to promote the vascular effects of this toxin. In this study we evaluated early effects of LeTx in highly vascularized lung tissue in order to identify transcriptional changes pointing to new potential mechanisms of LeTx-induced vascular effects including vascular leakage and pulmonary edema. Although this study was designed to identify the earliest LeTx-induced gene expression changes, other transcripts that are modulated at late time points may also be important in pathogenesis. Nevertheless, at least 8 of the identified differentially expressed transcripts are normally enriched in endothelial cells (Table 1, Table 2 and Table 4), including *Gpr182* and *Adrb2*, discussed in more detail below.

Though not an endothelial-specific gene *per se*, *Rgs2* is expressed in endothelial cells and was specifically induced by LeTx. *Rgs2* encodes Regulator of G-protein Signaling 2, a GTPase activating protein (GAP) critical for negative regulation of G (q) alpha receptors and control of vascular tone and blood pressure. Previously, the up-regulation of *Rgs2* was observed in the hearts of Swiss Webster mice 48 h post-inhalational Ames spore challenge [48]. We observed significant up-regulation of *Rgs2 in* A/J mice due to LeTx treatment alone. Mice deficient in this gene exhibit hypertension and decreased cGMP-mediated vascular relaxation [36,49], indicating that modulating vascular smooth muscle homeostasis is a unique role for this GAP. Elevated expression of *Rgs2* would thus be predicted to result in vascular relaxation and may contribute to LeTx-mediated animal death since lowered mean arterial blood pressure occurs early following LeTx infusion and predicts non-survivors of systemic LeTx exposure [6,50]. Interestingly, *Rgs2* was originally cloned as an early response gene up-regulated in activated T cells [51]. Given the lymphocyte gene signature also observed in this study, it is also possible that elevated *Rgs2* could be occurring primarily in T lymphocytes (see further discussion below). Further studies to assess expression of *Rgs2* in isolated vascular tissue from LeTx-treated animals will be needed to clarify this point. An interesting study would be to determine whether *Rgs2* deficient mice exhibit enhanced resistance to LeTx-mediated death.

Another important LeTx-mediated expression change observed was down-regulation of the endothelial-enriched gene *Gpr182* encoding the adrenomedullin receptor (ADMR). Adrenomedullin is a potent blood vessel-secreted vasoactive peptide hormone [33] that can suppress apoptosis of endothelial cells [52] and prevent bleomycin-induced lung injury when administered intratracheally to mice [53]. Interestingly, a previous study found dramatic up-regulation of *Adm*, the gene coding for adrenomedullin, in the hearts of Ames spore challenged mice [48]. Whether the *Gpr182* gene product is a *bona-fide* receptor for adrenomedullin is controversial, however. Binding of adrenomedullin to COS-7 cells transfected with rat or human ADMR was not detectable [54]; however, siRNA-mediated silencing of ADMR expression in both HUVEC and mouse lung endothelial cells demonstrated that autocrine effects of adrenomedullin in these cells were mediated through ADMR [55]. 

Expression of a second endothelial-enriched gene, adrenergic β receptor 2 (*Adrb2*) was modulated by LeTx treatment. Adrenergic β receptor 2 agonists have been demonstrated to participate in up-regulation of active Na^+^ transport *in vitro* [56]. More recently, up-regulation of alveolar fluid clearance due to increased active Na^+^ transport has been demonstrated *in vivo* [35,57]. Additionally, C57BL/6 *Adrb2* knockout mice have severely impaired ability to clear alveolar fluid when compared to wild type mice [34]. In our study, this gene was significantly down-regulated in A/J mice treated with LeTx; supporting that proper *Adrb2* function could be necessary for clearance of LeTx associated pulmonary edema. Further studies, such as exploring the effects of adrenergic β receptor 2 agonists on LeTx treated mice or evaluation of *Adrb2* deficient mice for enhanced susceptibility to LeTx-mediated death, are needed to investigate this possibility.

Our study also revealed a group of genes associated with cellular structure. These genes code for tubulin, actin, keratin, and other intermediate filaments that are involved with both inter- and intra-cellular architecture and may contribute to vascular leakage in the lungs [58]. Consistent with a previous study that observed the formation of actin stress fibers upon LeTx treatment [14], we observed the up-regulation of actin filament associating protein 1, or *Afap1*. Additionally, the down-regulation of *Krt79*, which codes for keratin, a component of desmosomes, is consistent with the possibility that the disruption of intercellular junctions may lead to vascular leakage. 

While the primary goal of this study was to identify new candidate pathways that may promote vascular leakage and pulmonary edema in LeTx-treated mice, additional groups of genes for other processes were also identified. One such identified process that was enriched among differentially expressed genes in A/J mice was cell death. Although endothelial cell death may not be the major cause of vascular/capillary leakage, cell death in other cell types due to exposure to LeTx may contribute to increased *B. anthracis* virulence. In this study we observed a down-regulated myeloid gene signature in A/J mice treated with LeTx. Since LeTx is known to kill macrophages [59,60] and host myeloid cell death may be essential for successful *B. anthracis* infection [61], this myeloid related gene signature is of interest. Genes expressed specifically in macrophages (*Mrc1*, *Csf2ra*, *Ccl6*, and *Sirpb1a*), neutrophils (*Ccl6*, *Sirpb1a*, and *Lcn2*) and other myeloid cells were observed. 

Products of another observed group of differentially expressed immune-related genes participate in neutrophil chemoattraction. Although neutrophilia is induced in the circulation by LeTx, margination of neutrophils into tissues is conspicuously absent in such animals [7]. Barson *et al*. observed the suppression of chemokine producing gene expression *in vitro* after 2 hours of LeTx exposure [62]. Furthermore, LeTx has been shown to inhibit neutrophil chemotaxis in a direct manner by a process that impairs actin filament assembly [13]. The present study suggests that LeTx may also impair neutrophil chemotaxis in whole lung tissue through additional mechanisms including transcriptional inhibition of neutrophil chemoattractants and transcriptional down-regulation of urokinase-type plasminogen activator (CD87/uPAR), encoded by *Plaur*. This gene product promotes trans-endothelial migration of neutrophils [44]. Mice deficient in uPAR demonstrated severely impaired neutrophil migration into lungs and impaired host defense against pulmonary infection with *Psuedomonas aeruginosa* [46] and Pneumococcal pneumonia [45]. 

Interestingly, a group of 15 differentially expressed genes, 12 in A/J mice and 3 in B6 mice, related to tumor regulation/angiogenesis was observed. Considering the unique cytoplasmic translocation method utilized by LF via PA [41], the specificity of the PA/receptor interaction and innate anti-angiogenic properties of LF, and the over-expression of the anthrax toxin receptor TEM-8 in tumor endothelium [63], it is not surprising that some investigators have begun to explore the use of LeTx as a method of tumor therapy [32,42,43]. Disrupting angiogenesis is a possible means by which LeTx achieves tumor regulation. The down-regulation of pro-angiogenic genes in our study such as *Gpr182* [55], *P2ry13* [64] and *Plaur* [65] is consistent with this. 

Unexpectedly, the most prominent LeTx-induced differentially expressed gene signature, which was found in both A/J and B6 mice, involved enhanced expression of multiple lymphoid genes, including genes encoding the canonical antigen receptors of both T and B lymphocytes. Ingenuity Pathways Analysis revealed significant enrichment of differentially expressed genes suggesting both quantity and activation of lymphocytes. Our histologic analysis of lung tissue revealed lungs devoid of lymphocytic infiltrates, in agreement with other studies demonstrating lack of immune cell infiltrates in lungs even at late time points following systemic LeTx exposure [52]. Because 6 h is an insufficient length of time for T or B cell proliferation in response to any stimulus, these prominent transcriptional changes could either represent a prelude to proliferation of lymphocytes already present in the lungs/lung vasculature or LeTx-mediated lymphocyte activation. Indeed, CD69 transcripts were elevated in LeTx-treated lungs of A/J mice at this early time point. In contrast, several groups have demonstrated that LeTx instead impairs lymphocyte proliferation and activation [11,12,66,67]. However, all of these studies either examined responses of isolated lymphocytes *in vitro* or examined restimulation of lymphocytes previously exposed to LeTx. One interesting study demonstrated that LeTx has a mitogenic effect on T cells, resulting in their proliferation [68]. This mitogenic effect was shown to be mediated indirectly through a soluble factor secreted by monocytes, and the proliferating cells had not been stimulated through the T cell receptor. The effect could not have been mediated by endotoxin contamination of LeTx components because T cell proliferation did not occur in the presence of PA or LF alone and also failed to occur in the presence of PA plus an inactive mutant of LF. The results of the present study lead to speculation that a similar mitogenic effect may be occurring *in vivo* in LeTx-treated mice, resulting in initial LeTx-mediated lymphocyte activation. Such an abnormal activation event could easily lead to an anergic state, rendering lymphocytes unresponsive to further stimulation. Interestingly, pharmacological treatment with interleukin-2 (IL-2) causes a vascular leakage syndrome [69,70], and rapid, systemic mitogenic T cell proliferation may well be accompanied by a burst of systemic IL-2.

## 5. Conclusions

This study has identified new directions for the investigation of LeTx-induced vascular effects, pulmonary edema and animal death. Further investigation of vascular G (q) alpha receptors, impaired adrenomedullin-mediated vascular protection, adrenergic β receptor 2-mediated pulmonary fluid clearance, and mitogenic T lymphocyte activation as possible mechanistic pathways promoting LeTx-induced death are warranted.
